# Regulatory Mechanisms of Cell Polyploidy in Insects

**DOI:** 10.3389/fcell.2020.00361

**Published:** 2020-05-29

**Authors:** Dani Ren, Juan Song, Ming Ni, Le Kang, Wei Guo

**Affiliations:** ^1^State Key Laboratory of Integrated Management of Pest Insects and Rodents, Institute of Zoology, Chinese Academy of Sciences, Beijing, China; ^2^CAS Center for Excellence in Biotic Interactions, University of Chinese Academy of Sciences, Beijing, China; ^3^College of Life Sciences, Hebei University, Baoding, China

**Keywords:** endocycle, juvenile hormone, 20-hydroxyecdysone, mitotic/endocycle switch, cell cycle

## Abstract

Polyploidy cells undergo the endocycle to generate DNA amplification without cell division and have important biological functions in growth, development, reproduction, immune response, nutrient support, and conferring resistance to DNA damage in animals. In this paper, we have specially summarized current research progresses in the regulatory mechanisms of cell polyploidy in insects. First, insect hormones including juvenile hormone and 20-hydroxyecdysone regulate the endocycle of variant cells in diverse insect species. Second, cells skip mitotic division in response to developmental programming and conditional stimuli such as wound healing, regeneration, and aging. Third, the reported regulatory pathways of mitotic to endocycle switch (MES), including Notch, Hippo, and JNK signaling pathways, are summarized and constructed into genetic network. Thus, we think that the studies in crosstalk of hormones and their effects on canonical pathways will shed light on the mechanism of cell polyploidy and elucidate the evolutionary adaptions of MES through diverse insect species.

## Introduction

**FIGURE 1:**
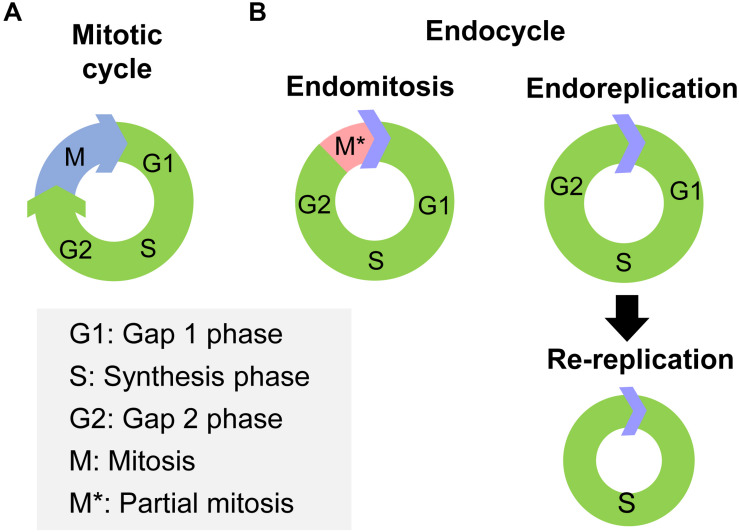
The processes of mitotic cycle and endocycle. **(A)** The mitotic cycle comprises Gap 1 phase (G1), synthesis phase (S), Gap 2 phase (G2), and mitosis (M) phase. **(B)** The endocycle have two variations, endomitosis and endoreplication. Endomitosis consists of G1, S, and G2 phases and only a partial M phase. Endoreplication consists of repeated successions of S to G phase that completely skips mitosis phase. Re-replication is a special type of endoreplication, in which DNA replication is initiated multiple times at individual origin of replication within the same S phase.

Cell polyploidy generated by endocycle is a cell cycle variant that undergoes multiple rounds of nuclear genome duplication in the absence of cell division (Zielke et al., 2013). In canonical mitotic cell cycles, cells pass through Gap 1 phase (G1), synthesis phase (S), Gap 2 phase (G2) and end in mitosis (M), thus the genetic materials of the mother cell are duplicated and delivered to two daughter cells (Figure 1A). However, in endocycles, cells increase their genomic DNA content without cell division (Lee et al., 2009). Two variations of endocycle are endoreplication and endomitosis (Zielke et al., 2013). Endoreplication consists of successive Synthesis phase to Gap phase that completely skips mitosis (Lee et al., 2009; Maldonado et al., 2019; Figure 1B). Cells that undergoes endoreplication result in a single, enlarged, polyploid nucleus (Calvi, 2013). A special type of endoreplication is re-replication, in which DNA is initiated multiple times at individual origins of replication within a single S phase, provoking site-specific replication of an unique sequence (Lee et al., 2009; Figure 1B). Thus highly amplified and underreplicated DNA sequences at different loci form supersized multifiber-like chromosomes called polytene chromosomes (Stormo and Fox, 2017). While, endomitosis consists of G1, S, G2, and partial M phases, which produces cells with a single giant nucleus, which may subsequently fragment into a multinuclear appearance (Zielke et al., 2013; Figure 1B).

The biological purposes of endocycle include tissue growth, blood-brain barrier formation, immune response, nutrient support, and conferring resistance to DNA damage based on tiss types and developmental stages (Lilly and Spradling, 1996; Zhang et al., 2014; Lynch and Marinov, 2015; Bretscher and Fox, 2016; Wu et al., 2018; Zülbahar et al., 2018; Maldonado et al., 2019). Endocycle is considered a low-cost strategy to increase the cell and/or tissue size and efficiently produce massive molecules needed for specific functions, which avoids spending more time, materials, and energy to go through complete mitotic cycles (Edgar and Orr-Weaver, 2001; Lynch and Marinov, 2015; Maldonado et al., 2019). Thus, polyploidy cells have advantages in the adaptation of organisms to environment; however, they can also have detrimental effects on fertility and fitness owing to genomic instability, mitotic and meiotic abnormalities, and gene expression and epigenetic changes (Zhimulev, 1996; Van de Peer et al., 2017). On the other hand, unpredicted cell polyploidy is closely related to the development and progression of cancer in mammals (Storchova and Pellman, 2004; Davoli and de Lange, 2011; Fox and Duronio, 2013; Coward and Harding, 2014). Clinically, endocycle and polyploidy have been observed in cancerous tissues, with their occurrence ranging from 11% in stomach carcinoma to 54% in liver adenocarcinoma (Storchova and Pellman, 2004; Shu et al., 2018). Therefore, these findings provided important clues for revealing the essential roles of polyploidy in normal development and tissue, homeostasis, as well as the relationship between polyploidy and the progression of cancer in vertebrate animals.

Previously, some scientists have summarized the key genes that play a role in cell polyploidy and mainly focus on the cell division arrest. The interactive regulation of E2f1, cyclin E, and cyclin-dependent kinase (CDK) promote endocycle occurrence through bypassing many of the processes of mitosis in insects and mammals (Zielke et al., 2013). And the biological purposes of cell polyploidy in many different organisms are generally discussed (Edgar and Orr-Weaver, 2001; Lee et al., 2009; Fox and Duronio, 2013). Insects as the biggest groups in invertebrates have common phenomena of cell polyploidy; however, a thorough summary of previous studies involving in more extensive insect species is lacking except in *Drosophila*. Here we review the cells types with polyploidy and recent progresses in the regulatory mechanisms of cell polyploidy in insects with special emphasis on the aspects of hormones action and environmental stimuli. In addition, we constructed a regulatory network based on previous and recent reported signaling pathways involved in MES. Finally, we proposed a promising MES study system and potential directions in cell polyploidy study in insects.

## Polyploid Cells in Insects

Polyploidy is commonly observed in highly metabolic tissues or cells in both complete and incomplete metamorphosis insects (Table 1). The fat body, midgut, muscle, Malpighian tubules, and nurse cells are the most reported tissues or cells with polyploidy (Zhimulev, 1996; Lee et al., 2009; Wu et al., 2018; Maldonado et al., 2019). The ploidy levels ranged from 4C in the fat body cells of *Locusta migratoria* to 2048C in the salivary glands of *Drosophila* and varied from 4C to 64C in different cells of single tissue (Hammond and Laird, 1985; Guo et al., 2014). Most of these polyploid tissues or cells can rapidly and massively synthesize proteins upon necessity during development, reproduction, immunity, flight, and other life activities (Ray et al., 2009; Fox and Duronio, 2013; Rangel et al., 2015; Wu et al., 2016).

**TABLE 1:** Tissues or cells with polyploidy in insects.

Order	Species	Tissue/cell type	Function	References
Diptera	*Drosophila melanogaster*	Brain/subperineurial glial cells	Blood-brain barrier	Unhavaithaya and Orr-Weaver, 2012; Zülbahar et al., 2018
		Ovary/nurse cells	Provide vast amounts of maternal messages and products for the developing oocyte	Royzman et al., 2002
		Ovary/follicle cells	Oocyte maturation and egg chamber development/eggshell gene amplification, DNA damage resistance	Calvi and Spradling, 1999
		Papillar cells	Repress the apoptotic response to DNA damage	Zhang et al., 2014; Bretscher and Fox, 2016
		Salivary glands	Synthesis and secretion glue proteins	Hammond and Laird, 1985; Buntrock et al., 2012
		Fat body	–	Juhasz and Sass, 2005
		Hindgut/rectal papillae	–	Fox and Duronio, 2013
		Trachea	–	Fox and Duronio, 2013
		Malpighian tubules	Absorb water, solutes, and wastes and excrete them as nitrogenous compounds	Lamb, 1982
		Renal tubules	–	Fox and Duronio, 2013
		Epidermis	–	Fox and Duronio, 2013
		Mechanosory bristle/shaft cells	–	Audibert et al., 2005
		Mechanosory bristle/socket cells	–	Audibert et al., 2005
	*Calliphora erythrocephala*	Ovary/nurse cells	–	Ribbert, 1979
	*Aedes aegypti*	Midgut	Immune response	Ray et al., 2009; Maldonado et al., 2019
	*Culex pipiens*	Midgut	Immune response	Ray et al., 2009; Maldonado et al., 2019
	*Anopheles gambiae*	Ovary/nurse cells	–	Zhimulev, 1996
	*Anopheles albimanus*	Ovary/nurse cells	–	Zhimulev, 1996
	*Chironomus tentans*	Salivary glands	–	Zhimulev et al., 2004
Orthoptera	*Locusta migratoria*	Fat body (female)	Massive synthesis of vitellogenin	Guo et al., 2014; Wu et al., 2016; Wu et al., 2018
		Fat body (male)	–	Ren et al., 2019
		Fat body-like tissue	–	Ren et al., 2019
		Ovary/follicle cells	Oocyte maturation and egg chamber development	Wu et al., 2016; Wu et al., 2018
Coleoptera	*Tribolium castaneum*	Midgut/intestinal stem cells	–	Parthasarathy and Palli, 2008
Lepidoptera	*Ephestia kuehniella*	Malpighian tubules	–	Buntrock et al., 2012
		Silk glands	High silk production	Buntrock et al., 2012
		Wing epithelium	Increase cell size	Edgar and Orr-Weaver, 2001
	*Spodoptera frugiperda*	Ovary	–	Meneses-Acosta et al., 2001
Hymenoptera	*Apis mellifera*	Malpighian tubules	–	Rangel et al., 2015
		Brain	–	Rangel et al., 2015
		Stinger	–	Rangel et al., 2015
		Leg	–	Rangel et al., 2015
		Thoracic muscle	–	Rangel et al., 2015
		Flight muscle	–	Rangel et al., 2015
	*Bombus terrestris* L.	Mandibular muscle (haploid male)	Keep pace with females in terms of muscular metabolic activity and efficiency	Aron et al., 2005
		Thoracic muscle (haploid male)	Keep pace with females in terms of muscular metabolic activity and efficiency	Aron et al., 2005
		Leg muscles (haploid male)	Keep pace with females in terms of muscular metabolic activity and efficiency	Aron et al., 2005
	*Solenopsis invicta*	Whole body	Body size and behavior	Scholes et al., 2013
	*Pogonomyrmex badius*	Whole body	Body size and behavior	Scholes et al., 2013
	*Camponotus floridanus*	Whole body	Body size and behavior	Scholes et al., 2013
	*Atta texana*	Whole body	Body size and behavior	Scholes et al., 2013

In the fruit fly *Drosophila melanogaster*, larval growth is achieved primarily via endoreplication (Edgar and Nijhout, 2004). Most *Drosophila* larval tissues are composed mainly of polyploid cells including the salivary glands, fat body, germline cells, subperineurial glia, epidermis, gut, trachea, and Malpighian or renal tubules (Lee et al., 2009). The giant salivary gland cells undergo about 10 endocycles, resulting in polytene chromosomes (Hammond and Laird, 1985; Maldonado et al., 2019). In adult *Drosophila*, the maximum polyteny level is 64C in the midgut and 256C in Malpighian tubules, respectively (Lamb, 1982). *Drosophila* papillar cells become polyploid and naturally accumulate broken acentric chromosomes but do not apoptose/arrest the cell cycle, thus they can divide and survive despite high levels of DNA breakage (Zhang et al., 2014; Bretscher and Fox, 2016). The fat body generates polytenic cells through re-replication (Juhasz and Sass, 2005). In the female germline, nurse cells become polyploid during oogenesis, enabling them to provide vast amounts of maternal messages and products for the developing oocyte, whereas the somatic follicle cells that surround the egg undergo only three endocycles from stages 7–9 to reach a ploidy level of 16C, which facilitates the high levels of functional gene expression needed for reproduction (Calvi and Spradling, 1999; Royzman et al., 2002). Interestingly, the subperineurial glia expand enormously and become polyploid undergo both endoreplication and endomitosis, allowing it to accommodate growing neurons, while simultaneously maintaining the blood-brain barrier, which otherwise would be disrupted through cell division (Unhavaithaya and Orr-Weaver, 2012; Zülbahar et al., 2018). During *Drosophila* pupal development, shaft and socket cells that form parts of the mechanosory bristle undergo two or three endocycles to produce cells with 8C or 16C DNA (Audibert et al., 2005). Therefore, the extensive cell polyploidy in *Drosophila* variant tissues and developmental stages displays the cellular potentials to remodel for various functions.

Polyploidy cells are also found in variant complete metamorphosis insect species. In mosquitoes, polyploidy cells arise in the anterior and posterior midgut of *Aedes aegypti*, yet only in anterior midgut *of Culex pipiens* during larval development (Ray et al., 2009). These polyploid midgut cells facilitate the fast production of immune proteins in a process known as priming (Maldonado et al., 2019). Polyploid chromosomes are formed in ovarian nurse cells of *Anopheles albimanus* and polytene chromosomes are formed in ovarian nurse cells of *Anopheles gambiae* and salivary glands of *Chironomus tentans* (Zhimulev, 1996; Zhimulev et al., 2004). In the *Calliphora erythrocephala*, nurse cells also developed polytene chromosomes post inbreeding and artificial selection (Ribbert, 1979). In the honey bee *Apis mellifera*, the Malpighian tubules is the most highly polyploid secretory cells, and the brain, stinger, leg, thoracic muscle, and flight muscle also generate polyploid cells (Rangel et al., 2015). In the bumble bee *Bombus terrestris L.*, cells in mandibular, thoracic, and leg muscles of the haploid male undergo one round of endoreduplication to become functionally diploid and get comparable size and function to those of the diploid female (Aron et al., 2005). Ploidy levels between and among worker castes of four highly polymorphic ant species, red imported fire ant *Solenopsis invicta*, Florida harvester ant *Pogonomyrmex badius*, Florida carpenter ant *Camponotus floridanus*, and Texas leaf-cutting ant *Atta texana*, are positively related to worker size, suggesting that worker task performance might benefit from endoreplication of certain tissues (Scholes et al., 2013). In the red flour beetle *Tribolium castaneum* larval stages, intestinal stem cells (ISC) conduct endoreplication for adult midgut polyploidic epithelium formation (Parthasarathy and Palli, 2008). In the flour moth *Ephestia kuehniella*, cells of Malpighian tubules and silk glands undergo endocycle through all larvae instars, and even, in the last instar, larvae nuclei are polyploid with a high DNA content, provoking a branched nucleus (Buntrock et al., 2012). In *Spodoptera frugiperda* sf9 ovarian cells, cell cycle arrests in G2/M phase to generate polynucleated cells (Meneses-Acosta et al., 2001).

In the migratory locust, *Locusta migratoria*, an incomplete metamorphosis insect species, fat body cells of female adults undergo extensive DNA replication to produce from 4C to 64C polyploidy cells during vitellogenesis. These polyploidy cells support the rapidly massive synthesis of vitellogenin, the main proteins in egg maturation, for dozens of oocytes in every gonadotrophic cycle. Besides, female locust follicle cells also undergo high levels of endoreplication during oocyte maturation to synthesize chorion protein for success chorionogenesis (Kimber, 1980; Guo et al., 2014; Wu et al., 2016, 2018). While in the male locust, the abdominal fat body (FB) undergoes endocycle and generates 4C to 8C polyploidy cells, while the fat body-like tissue (FLT) surrounding testis follicles is dominated by diploid cells instead of polyploidy cells (Ren et al., 2019). Thus, similar or same tissues in one species display different polyploidy levels between both genders or in different hemocal locations.

Both polyploid and polytene chromosomes originate by the endocycle. In the polytene chromosomes, the replicated sister chromatids remain attached and aligned and the chromosomes become visible. In polyploid cells, the replicated chromosome copies are dispersed and the chromosomes are not visible in the nucleus (Frawley and Orr-Weaver, 2015). Therefore, polytene chromosomes are specialized polyploid chromosomes. Transition between polytene and polyploid chromosome have been studied in *Drosophila* ovarian nurse cells (Bauer et al., 2012). Condensin II drives axial compaction and therefore force apart chromatids destroying a typical polytene chromosome structure, thus, polyploid chromosome are formed in the nurse cells (Bauer et al., 2012). It seems that polytene chromosomes formation are energy-saving to mainly skip duplicating heterochromatic regions, while polyploid chromosomes are advantageous to massively duplicate any gene upon necessity although its formation needs more energy (Frawley and Orr-Weaver, 2015; Stormo and Fox, 2017). Thus, we speculated that the use of different endoreplication strategies might indicate an adaptive trade-off between energy consuming in DNA amplification and the tissue demands for specific protein syntheses. Taken together, the extensive occurrence of cell polyploidy in variant insect species provide excellent study materials to decipher the regulatory mechanisms of endocycle in the light of evolution.

## Genetic Network in Endocycle

**FIGURE 2:**
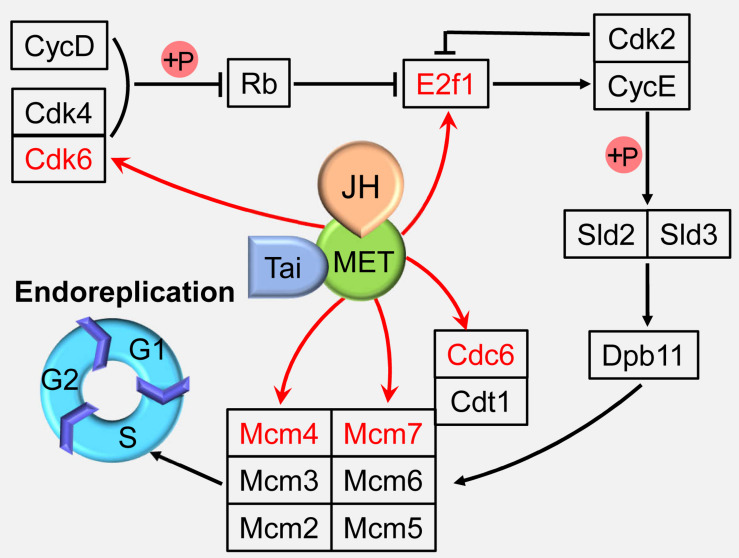
Juvenile hormone (JH) regulation of the endoreplication. JH-Met/Tai ligand-receptor complex directly activates the expressions of cell cycle genes including minichromosome maintenance proteins 4 and 7 (Mcm4 and 7), cell division cycle 6 (Cdc6), cyclin-dependent kinase 6 (Cdk6), and adenovirus E2 factor-1 (E2f1) to regulate endoreplication and cell polyploidy. Met, Methoprene-tolerant; Tai, Taiman; CycD, cyclin D; Cdk2 and 4, Cyclin-dependent kinase 2 and 4; Rb, retinoblastoma; CycE, cyclin E; Cdt1, Cdc10 protein-dependent transcript 1; Mcm2, 3, 5, and 6, minichromosome maintenance protein 2, 3, 5, and 6.

Although significantly different from the conventional cell cycle, endocycle uses regulatory pathways that also function in diploid cells (Lee et al., 2009; Calvi, 2013). Cyclin-dependent kinase (Cdk) 4/6 form an active complex with Cyclin D (CycD), phosphorylate and inactivate retinoblastoma protein (Rb), releasing transcription factor adenovirus E2 factor-1 (E2f1) from Rb-mediated repression (Swanson et al., 2015; Tigan et al., 2016). Cyclin E (CycE), which is activated by E2f1, together with cyclin-dependent kinase 2 (Cdk2), triggers S-phase by phosphorylating Sld2 and Sld3, which then recruit Dpb11 to replication origins, thereby activating the Mini-chromosome maintenance (Mcm) 2–7 DNA helicase (Zielke et al., 2011; Tanaka and Araki, 2012; Bell and Kaguni, 2013). Together with the origin recognition complex and Cdt1 (Cdc10 protein-dependent transcript 1), Cdc6 loads Mcm proteins onto DNA at the origins of replication to facilitate the formation of stable prereplication complex in G1 phase, thereby licensing these sites to initiate DNA replication in S phase (Sun et al., 2013; Wu et al., 2016; Figure 2).

## Endocycle Governed by Insect Hormone

Juvenile hormone (JH) and 20-hydroxyecdysone (20E), as two dominant hormones that are involved in insect development, metamorphosis, and reproduction, play key roles in the regulation of cell polyploidy by binding to their respective nuclear receptors to initiate the expression of cell cycle genes (Fallon and Gerenday, 2010; Jindra et al., 2013; Yamanaka et al., 2013; Guo et al., 2014; Moriyama et al., 2016; Wu et al., 2016, 2018; Maldonado et al., 2019). Many studies have confirmed that the two hormones can regulate cell polyploidy in insects.

Juvenile hormone is a sesquiterpenoid produced by the corpora allata, and functions in cells by inducing the heterodimerization of its receptor, Methoprene-tolerant (Met) with Taiman (Tai). Met and Tai form a functional receptor to control insect development, metamorphosis, and reproduction (Jindra et al., 2013; Wang et al., 2017). The migratory locust, *Locusta migratoria* is the most well-studied insect species in the JH regulation on cell polyploidy. The adult fat body undergoes extensive DNA replication to highly polyploidy cells during vitellogenesis for successful reproduction under the regulation of JH (Nair et al., 1981; Oishi et al., 1985). In an effort to elucidate the mechanisms of JH action on female reproduction, digital gene expression profiling was employed to identify differentially expressed genes in JH-deprived fat bodies and those further treated with a JH analog (JHA). DNA replication pathway was identified as the top one “hit” after JHA treatment by Kyoto Encyclopedia of Genes and Genomes (KEGG) analyses (Guo et al., 2014). Further study revealed that Met binds directly on the E-box (CACGTG) or E-box-like (CACGCG) motifs in the promoter regions of Mcm4, Mcm7, and Cdc6 to activate the transcription of these genes, which promotes the endoreplication of fat body cells (Guo et al., 2014; Wu et al., 2016). In addition, JH-Met/Tai complex directly activates the transcription of Cdk6 and E2f1, and depletion of Cdk6 or E2f1 results in significantly decreased the cell polyploidy level, precocious mitotic entry for multinuclear appearance, and increased cell numbers in the fat body cells (Wu et al., 2018; Figure 2). These results indicate that JH is able to directly activate several cell cycle genes to enhance endoreplication process.

**FIGURE 3:**
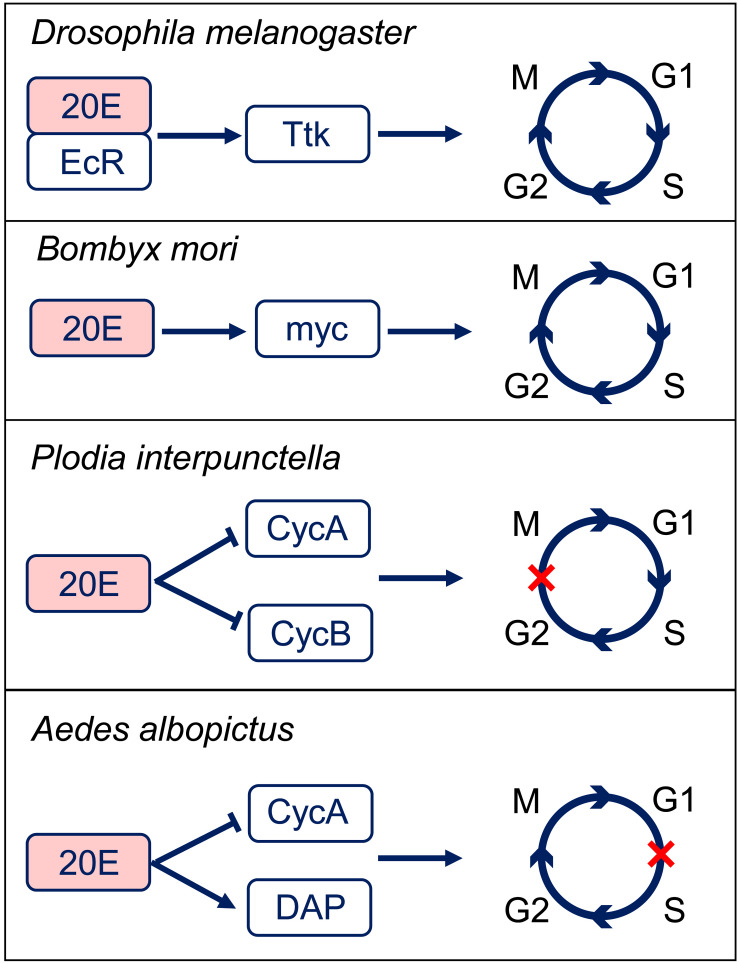
20-hydroxyecdysone (20E) regulation of the cell cycle progression. In *Drosophila melanogaster*, 20E-EcR positively regulates Tramtrack (Ttk) to transit the endocycle to mitotic cycle. In *Bombyx mori*, 20E promotes cell cycle progression through activation of c-Myc. In *Plodia interpunctella*, 20E induces an arrest of cells in G2 phase by decreasing the expressions of cyclin (Cyc) A and B. In *Aedes albopictus*, 20E application results in an accumulation of cells in G1 phase by decreasing CycA and increasing Dacapo (DAP) expresisons. EcR, ecdysone receptor.

The ecdysteroid is also involved in cell cycle regulation in a different way from JH. The major ecdysteroid in insects such as 20E not only promotes molting at juvenile stages but also affects lifespan, learning, stress-induced responses, sleep regulation, social interactions, and sexual behavior in adults (Yamanaka et al., 2013). 20E mediate the switch between endocycle and site-specific endoreplication by binding to the ecdysone receptor (EcR) (Maldonado et al., 2019). Importantly, 20E also regulates DNA replication and polyploidy during insect metamorphosis (Sun et al., 2008). Correlations between epidermal DNA synthesis and hemolymph ecdysteroid levels have been revealed in the tobacco hornworm *Manduca sexta* and *Calpodes ethlius* (Wielgus et al., 1979; Dean et al., 1980). In *Plodia interpunctella*, imaginal wing cells arrest in G2 phase post 20E treatment by inducing a sharp decrease in the levels of cyclin A and B expression (Mottier et al., 2004). In cell line C7-10 from the mosquito *Aedes albopicus*, 20E application resulted in cell arrest in the G1 phase (Gerenday and Fallon, 2004). Further studies suggest that 20E treatment increases the expression of Cyclin E-Cdk2 inhibitory protein DACAPO, while decreases the expression of cyclin A (Fallon and Gerenday, 2010). In the wing discs of silkworm *Bombyx mori*, 20E directly activates c-Myc transcription, which subsequently stimulates the expression of cell cycle core regulators, including cyclinA, cyclinB, cyclinD, cyclinE, Cdc25, and E2F1 genes to promote endocycle progression and cell polyploidization during metamorphosis (Moriyama et al., 2016; Figure 3). These studies indicate that 20E promotes cell proliferation in low titer and initiates cell cycle arrest in high titer (Koyama et al., 2004; Moriyama et al., 2016). Thus, actions of 20E on cell polyploidy vary in different insect species in a dose-dependent way.

However, the interactions between JH and 20E in cell polyploidy regulation are still largely unknown. Classically, 20E counteracts on the function of JH during molting and metamorphosis (Liu P. C. et al., 2018; Liu S. et al., 2018). Studies of JH and 20E actions on cell polyploidy focus mainly on the aspects of DNA reduplication enhancement and cell division arrest, respectively. JH and 20E may jointly coordinate the timing of DNA reduplication and cell division during MES process.

## Environment-Evoked Endocycle

In response to pathogen, wound, and aging, some tissue cells are re-programmed, exiting their mitotic cell cycle to differentiate into polyploidy cells (Zielke et al., 2013). In *Drosophila*, ovarian pseudonurse cells raised the frequency of polytene chromosomes in response to low temperature and protein-rich food (Mal’ceva et al., 1995). In *Anopheles albimanus*, the increase of cell DNA synthesis after challenged with different microorganism and *Plasmodium sp.* is considered as an adaptive immune response, which can be recalled quickly in next exposure to the same pathogens (Hernández-Martínez et al., 2013; Contreras-Garduño et al., 2015). In the adult *Drosophila* epidermis and hindgut, post-mitotic differentiated diploid cells respond to wounding by entering the endocycle, and this process is called wound-induced polyploidization (WIP) (Losick et al., 2016). In the honey bee *Apis mellifera*, endoreplication levels change with worker age in a tissue-specific manner, and there is a surprisingly significant decrease in cell ploidy in the leg and the thoracic muscles with aging (Rangel et al., 2015).

On the other hand, the mitotic cell cycle can be activated in some insects post stimuli. In *Aedes albopictus*, midgut generates an increase in regenerative cells after chemical and bacterial damages (Janeh et al., 2017). Besides, cell proliferation has been tested in *Aedes aegypti* midgut post viral infections and oxidative stress (Taracena et al., 2018). In *Drosophila*, starvation reduces E2f1 mRNA levels and blocks endocycling in salivary glands, whereas overexpression of E2f1 restores endocycles in starved fruit flies (Zielke et al., 2011). Inhibition of the target of rapamycin (TOR) in *Drosophila* prothoracic gland causes nutrient-dependent endocycle inhibition and developmental arrest (Ohhara et al., 2017). Thus, cell polyploidy displays adaptive plasticity to respond to unpredicted environment changes through remodeling the mitotic cycle to endocycle.

## Molecular Mechanisms of Mitosis/Endocycle Switch

Cells switch from the mitotic cycle to the endocycle response to developmental signals or environmental stimuli, and become polyploidy rather than proliferation. This intermediate process is defined as the mitosis/endocycle switch (MES). The thrombopoetin and the Notch pathways have been identified as the major regulators of the mitotic-to-endocycle switch. The thrombopoetin pathway acts during differentiation of megakaryocytes, and the Notch pathway acts during the oogenesis and differentiation of trophoblasts (Zimmet and Ravid, 2000; Deng et al., 2001; López-Schier and St Johnston, 2001). Besides, the JNK pathway is also demonstrated to be required to promote mitosis prior to the transition, independent of the cell cycle components acted on by the Notch pathway (Jordan et al., 2006). In follicle cells, the Notch pathway stops proliferation and promotes a switch from the mitotic cycle to the endocycle (Sun and Deng, 2007). In contrast to Notch signal, Hedgehog (Hh) signal appears to promote follicle cell proliferation (Zhang and Kalderon, 2000).

**FIGURE 4:**
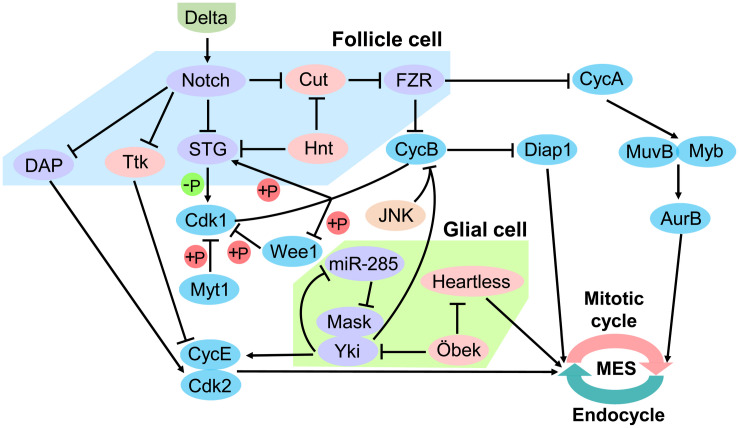
Regulation of mitotic/endocycle switch. In the *Drosophila* follicle cell, Notch signaling pathway is a key upstream regulator of the mitotic/endocycle switch (MES). Activated Notch protein by the ligand Delta from oocytes terminates follicle cell proliferation and promotes MES by downregulating String (STG) and Dacapo (DAP) and upregulating FZR. In the *Drosophila* glial cell, Hippo signaling pathway is involved in MES. miR-285 suppresses Yki-Mask dimer activity by targeting Mask to downregulate cyclin E (CycE). Moreover, the N-terminal asparagine amidohydrolase homolog Öbek counteracts the activity of Yki and Heartless to limit endoreplication. FZR, Cdh1/Fizzy-related; Yki, Yorkie; Mask, Multiple Ankyrin repeats Single KH domain; Hnt, Hindsight; Ttk, Tramtrack; Cdk1 and 2, Cyclin-dependent kinase 1 and 2; Myt1, Myelin transcription factor 1; CycA and B, cyclin A and B; Diap1, *Drosophila* inhibitor-of-apoptosis protein 1; AurB, AuroraB.

The Notch signaling pathway in *Drosophila* follicle epithelium is a key upstream regulator of the MES. The ligand Delta expressed by oocytes activates the Notch receptor in follicle cells (Deng et al., 2001). Notch activity leads to downregulation of String (STG) and Dacapo (DAP), and upregulation of Cdh1/Fizzy-related (FZR) (Jordan et al., 2006). STG is a G2/M regulator Cdc25 phosphatase that removes inhibitory phosphates from Cdk1, allowing it to associate with CycA and CycB and initiate mitosis (Zielke et al., 2013). CycB-Cdk1 in return activates the STG by phosphorylation, which creates a positive feedback loop (Zielke et al., 2013). The activity of CycB-Cdk1 is further inactivated through phosphorylation (Welburn et al., 2007). Cdk1 can be phosphorylated on Tyr15 by Wee1 kinase, and on both Thr14 and Tyr15 by Myelin transcription factor 1 (Myt1) kinase (Parker and Piwnica-Worms, 1992; Mueller et al., 1995). Interestingly, Wee1 is phosphorylated and thereby inactivated by CycB-Cdk1, which creates a double negative feedback loop (Tang et al., 1993). CycA/CDK directly activates the Myb-MuvB (MMB) complex to induce transcription of a battery of cell cycle genes required for mitosis. AuroraB (AurB) is an MMB regulated gene, and knockdown of AurB and other subunits of the chromosomal passenger complex (CPC) induced endoreplication (Rotelli et al., 2019). Down-regulating of the CycE/CDK complex inhibitor DAP releases CycE/Cdk2, thus blocks S phase initiation (Zielke et al., 2013). FZR is a regulator of the APC ubiquitination complex that degrade CycA and CycB, thereby reinforcing the block to mitosis (Zielke et al., 2013). FZR is negatively regulated by the homeodomain gene Cut, but Cut does not seem to regulate String (Stg) (Sun and Deng, 2005, 2007). String/Cdc25 and the transcription factor Cut are repressed by a zinc-finger transcription factor Hindsight (Hnt) (Sun and Deng, 2007). However, during oogenesis, Notch in *Drosophila* follicle cells is down-regulated and cooperate with Tramtrack (Ttk), a transcription factor that induces endocycle exit and entry in site-specific endoreplication (Sun et al., 2008). In the *Drosophila* bristle lineage, Ttk downregulates Cyclin E expression and is probably involved in the exit of the cells from the cell cycle (Audibert et al., 2005; Figure 4).

Hippo pathway in the *Drosophila* nervous system has been reported to be involved in the MES. Yki, an essential transcriptional activator of Hippo pathway, is required to establish the correct ploidy and is partly post-transcriptionally regulated miR-285. miR-285 is an upstream regulator of the Hippo pathway, which can directly target Yki cofactor Multiple Ankyrin repeats Single KH domain (Mask) to suppress Yki activity and down-regulates the expression of its downstream target cyclin E (CycE). Disturbance of CycE expression in subperineurial glial cell (SPG) causes abnormal endoreplication, which leads to aberrant DNA ploidy and defective septate junctions (Li et al., 2017). Moreover, the N-terminal asparagine amidohydrolase homolog Öbek counteracts the activity of Yki, as well as the activity of the fibroblast growth factor (FGF) receptor Heartless in glial cells to limit endoreplication and the consequent appearance of extra nuclei. However, other dividing cells are not affected by alteration of Öbek expression (Zülbahar et al., 2018). Cooperative activation of Yki and JNK upregulates *Drosophila* inhibitor-of-apoptosis protein 1 (Diap1) prevents mitotic entry by downregulating G2/M cyclin CycB, thereby inducing endoreplication (Cong et al., 2018). The protein level of *Drosophila* CycB is mediated by ubiquitination-mediated degradation, and Diap1 contains a RING domain and acts as an E3 ligase that could cause ubiquitination-mediated degradation of CycB in either direct or indirect manner (Xu et al., 2009; Shabbeer et al., 2013; Cong et al., 2018; Figure 4).

A recent study in the migratory locust *Locusta migratoria* reported that FB cells undergo endocycle and become polyploidy, while FLT cells undergo rounds of mitotic cycle and dominated by diploid cells at the same developmental stages during male adult maturation. FB-FLT cells displayed different cell cycle strategy at the same developmental stages, avoiding the interference of developmental programming. Further analyses of comparative transcriptomes of FB-FLT provided valuable candidate cell cycle genes and transcription factors to investigate the molecular mechanism of MES (Ren et al., 2019). Probably, the FB-FLT of locust is one of novel promising systems to study MES.

The developmental signals and environmental stimuli can affect the performance of the MES. In endocycling cells, promoters of pro-apoptotic genes are silenced rendering them insensitive to DNA damage (Mehrotra et al., 2008). Starvation induces the pause of the M/E switch in *Drosophila* follicle cells, blocking the entry of egg chambers into vitellogenesis. Paused MES is induced by a reduction of insulin signal and involves a previously unknown crosstalk among FoxO, Cut, and Notch. FoxO is dispensable for the normal M/E switch. However, in paused MES, FoxO activates Cut expression cell-autonomously in follicle cells (Jouandin et al., 2014). Besides, MES regulation may vary in different tissues or cell types. The mitotic cyclins in endocycling follicle cells are regulated only by ubiquitin-dependent degradation, whereas in salivary glands, transcription of mitotic cyclins is terminated concomitantly with up-regulation of Fzr (Zielke et al., 2008; Maqbool et al., 2010). The suppression of mitotic regulators in endoreplicating salivary glands is mediated, at least in part, by the transcriptional repressor E2F2 (Zielke et al., 2011). Besides, Notch controls the switches of different cell cycle programs in follicle cells, yet the nurse cell endocycles are normal in Notch mutant clones (Lilly and Duronio, 2005).

In brief, *Drosophila* follicle cells and glial cells are two well-studied systems and locust FB-FLT would be a novel promising system to investigate the molecular mechanisms of MES (Sun and Deng, 2007; Li et al., 2017; Cong et al., 2018; Zülbahar et al., 2018; Ren et al., 2019). Developmentally controlled MES were regulated by Notch and Hippo signaling pathway; however, the environmental-stimulated MES have probable specific mechanisms which are condition-dependent.

## Prospects

Insects are the best models to study regulatory mechanisms of polyploidy, compared to vertebrates and plants. First, polyploidization can be observed at larvae and adult stages in both complete and incomplete metamorphosis insects. Second, cells in different tissues of insects undergo endocycle during particular developmental stages or response to environmental stimuli.

Insect hormones play critical roles on cell polyploidy progression. JH promotes cell polyploidization by directly upregulating several DNA replication genes (Guo et al., 2014; Wu et al., 2016, 2018). However, whether JH also acts on cell division genes remains to be studied. Besides, the role of 20E played in cell cycle regulation is unclear, and the target genes of 20E in cell cycle process are also largely unknown. In some processes, JH plays a critical role in defining the action of 20E, and 20E also affects the function of JH (Jia et al., 2017; Liu P. C. et al., 2018; Liu S. et al., 2018). In addition, JH and 20E are reported to regulate MES-related genes and miRNA (Gerenday and Fallon, 2011; Dong et al., 2015; Wang et al., 2016; Song et al., 2019). Therefore, the crosstalk of JH and 20E in cell polyploidy regulation through MES need to be further investigated to clarify their biological functions and adaptive mechanisms to developmental programming and environmental changes.

Because MES is regulated by several canonical pathways, further studies should be conducted to decipher the relationships of these pathways and their interactions orchestrated by hormones. In addition, we attempt to answer whether these pathways are conserved in the regulation on MES in different tissue cells and diverse insect species. Therefore, studies of cell cycle genes and related pathways will shed light on the evolution of cell polyploidy and provide more target genes for pest control.

## Author Contributions

LK, WG, and DR designed the research. DR, JS, and MN collected the references. DR, JS, WG, and LK wrote the manuscript.

## Conflict of Interest

The authors declare that the research was conducted in the absence of any commercial or financial relationships that could be construed as a potential conflict of interest.

## References

[B1] Aron S. de Menten L. Van Bockstaele D. R. Blank S. M. Roisin Y. (2005). When hymenopteran males reinvented diploidy. *Curr. Biol*. 15 824–827. 10.1016/j.cub.2005.03.017 15886099

[B2] Audibert A. Simon F. Gho M. (2005). Cell cycle diversity involves differential regulation of Cyclin E activity in the *Drosophila* bristle cell lineage. *Development* 132 2287–2297. 10.1242/dev.01797 15829522

[B3] Bauer C. R. Hartl T. A. Bosco G. (2012). Condensin II promotes the formation of chromosome territories by inducing axial compaction of polyploid interphase chromosomes. *PLoS Genet*. 8:e1002873. 10.1371/journal.pgen.1002873 22956908 PMC3431300

[B4] Bell S. P. Kaguni J. M. (2013). Helicase loading at chromosomal origins of replication. *Cold Spring Harb. Perspect. Biol*. 5:a010124. 10.1101/cshperspect.a010124 23613349 PMC3660832

[B5] Bretscher H. S. Fox D. T. (2016). Proliferation of double-strand break-resistant polyploid cells requires *Drosophila* FANCD2. *Dev. Cell* 37 444–457. 10.1016/j.devcel.2016.05.004 27270041 PMC4901310

[B6] Buntrock L. Marec F. Krueger S. Traut W. (2012). Organ growth without cell division: somatic polyploidy in a moth, *Ephestia kuehniella*. *Genome* 55 755–763. 10.1139/g2012-060 23057509

[B7] Calvi B. R. (2013). Making big cells: one size does not fit all. *Proc. Natl. Acad. Sci. U.S.A*. 110 9621–9622. 10.1073/pnas.1306908110 23723347 PMC3683767

[B8] Calvi B. R. Spradling A. C. (1999). Chorion gene amplification in *Drosophila*: A model for metazoan origins of DNA replication and S-phase control. *Methods* 18 407–417. 10.1006/meth.1999.0799 10455001

[B9] Cong B. Ohsawa S. Igaki T. (2018). JNK and Yorkie drive tumor progression by generating polyploid giant cells in *Drosophila*. *Oncogene* 37 3088–3097. 10.1038/s41388-018-0201-8 29535423

[B10] Contreras-Garduño J. Rodríguez M. C. Hernández-Martínez S. Martínez-Barnetche J. Alvarado-Delgado A. Izquierdo J. (2015). *Plasmodium berghei* induced priming in *Anopheles albimanus* independently of bacterial co-infection. *Dev. Comp. Immunol*. 52 172–181. 10.1016/j.dci.2015.05.004 26004500

[B11] Coward J. Harding A. (2014). Size does matter: why polyploid tumor cells are critical drug targets in the war on cancer. *Front. Oncol*. 4:123. 10.3389/fonc.2014.00123 24904834 PMC4033620

[B12] Davoli T. de Lange T. (2011). The causes and consequences of polyploidy in normal development and cancer. *Annu. Rev. Cell Dev. Biol*. 27 585–610. 10.1146/annurev-cellbio-092910-154234 21801013

[B13] Dean R. L. Bollenbacher W. E. Locke M. Smith S. L. Gilbert L. I. (1980). Haemolymph ecdysteroid levels and cellular events in the intermoult/moult sequence of *Calpodes ethlius*. *J. Insect Physiol*. 26 267–280. 10.1016/0022-1910(80)90073-6

[B14] Deng W. M. Althauser C. Ruohola-Baker H. (2001). Notch-Delta signaling induces a transition from mitotic cell cycle to endocycle in *Drosophila* follicle cells. *Development* 128 4737–4746. 11731454 10.1242/dev.128.23.4737

[B15] Dong D. J. Jing Y. P. Liu W. Wang J. X. Zhao X. F. (2015). The steroid hormone 20-Hydroxyecdysone up-regulates Ste-20 family serine/threonine kinase Hippo to induce programmed cell death. *J. Biol. Chem*. 290 24738–24746. 10.1074/jbc.M115.643783 26272745 PMC4598986

[B16] Edgar B. A. Orr-Weaver T. L. (2001). Endoreplication cell cycles: more for less. *Cell* 105 297–306. 10.1016/s0092-8674(01)00334-8 11348589

[B17] Edgar B. A. Nijhout H. F. (2004). “Growth and cell cycle control in *Drosophila*,” in *Cell Growth – Control of Cell Size.* Hall M. N. Raff M. Thomas G. (Cold Spring Harbor, NY: Cold Spring Harb Perspect Biol) 23–83.

[B18] Fallon A. M. Gerenday A. (2010). Ecdysone and the cell cycle: investigations in a mosquito cell line. *J. Insect Physiol*. 56 1396–1401. 10.1016/j.jinsphys.2010.03.016 20303973 PMC2918671

[B19] Fox D. T. Duronio R. J. (2013). Endoreplication and polyploidy: insights into development and disease. *Development* 140 3–12. 10.1242/dev.080531 23222436 PMC3513989

[B20] Frawley L. E. Orr-Weaver T. L. (2015). Polyploidy. *Curr. Biol*. 25 R353–R358. 10.1016/j.cub.2015.03.037 25942544

[B21] Gerenday A. Fallon A. M. (2004). Ecdysone-induced accumulation of mosquito cells in the G1 phase of the cell cycle. *J. Insect Physiol*. 50 831–838. 10.1016/j.jinsphys.2004.06.005 15350503

[B22] Gerenday A. Fallon A. M. (2011). Increased levels of the cell cycle inhibitor protein, dacapo, accompany 20-hydroxyecdysone-induced G1 arrest in a mosquito cell line. *Arch. Insect Biochem. Physiol*. 78 61–73. 10.1002/arch.20440 21928393 PMC3546116

[B23] Guo W. Wu Z. Song J. Jiang F. Wang Z. Deng S. (2014). Juvenile hormone-receptor complex acts on mcm4 and mcm7 to promote polyploidy and vitellogenesis in the migratory locust. *PLoS Genet*. 10:e1004702. 10.1371/journal.pgen.1004702 25340846 PMC4207617

[B24] Hammond M. P. Laird C. D. (1985). Chromosome structure and DNA replication in nurse and follicle cells of *Drosophila melanogaster*. *Chromosoma* 91 267–278. 10.1007/bf00328222 3920017

[B25] Hernández-Martínez S. Barradas-Bautista D. Rodríguez M. H. (2013). Differential DNA synthesis in *Anopheles albimanus* tissues induced by immune challenge with different microorganisms. *Arch. Insect Biochem. Physiol*. 84 1–14. 10.1002/arch.21108 23797988

[B26] Janeh M. Osman D. Kambris Z. (2017). Damage-induced cell regeneration in the midgut of *Aedes albopictus* mosquitoes. *Sci. Rep*. 7:44594. 10.1038/srep44594 28300181 PMC5353711

[B27] Jia Q. Liu S. Wen D. Cheng Y. Bendena W. G. Wang J. (2017). Juvenile hormone and 20-hydroxyecdysone coordinately control the developmental timing of matrix metalloproteinase-induced fat body cell dissociation. *J. Biol. Chem*. 292 21504–21516. 10.1074/jbc.M117.818880 29118190 PMC5766943

[B28] Jindra M. Palli S. R. Riddiford L. M. (2013). The juvenile hormone signaling pathway in insect development. *Annu. Rev. Entomol*. 58 181–204. 10.1146/annurev-ento-120811-153700 22994547

[B29] Jordan K. C. Schaeffer V. Fischer K. A. Gray E. E. Ruohola-Baker H. (2006). Notch signaling through tramtrack bypasses the mitosis promoting activity of the JNK pathway in the mitotic-to-endocycle transition of *Drosophila* follicle cells. *BMC Dev. Biol*. 6:16 10.1186/1471-213X-6-16 PMC1436016 16542414

[B30] Jouandin P. Ghiglione C. Noselli S. (2014). Starvation induces FoxO-dependent mitotic-to-endocycle switch pausing during *Drosophila* oogenesis. *Development* 141 3013–3021. 10.1242/dev.108399 24993942 PMC6514422

[B31] Juhasz G. Sass M. (2005). Hid can induce, but is not required for autophagy in polyploid larval *Drosophila* tissues. *Eur. J. Cell Biol*. 84 491–502. 10.1016/j.ejcb.2004.11.010 15900708

[B32] Kimber S. J. (1980). The secretion of the eggshell of *Schistocerca gregaria*: ultrastructure of the follicle cells during the termination of vitellogenesis and eggshell secretion. *J. Cell Sci*. 46 455–477. 7228915 10.1242/jcs.46.1.455

[B33] Koyama T. Iwami M. Sakurai S. (2004). Ecdysteroid control of cell cycle and cellular commitment in insect wing imaginal discs. *Mol. Cell Endocrinol*. 213 155–166. 10.1016/j.mce.2003.10.063 15062563

[B34] Lamb M. J. (1982). The DNA content of polytene nuclei in midgut and Malpighian tubule cells of adult *Drosophila melanogaster*. *Wilehm. Roux Arch. Dev. Biol*. 191 381–384. 10.1007/BF00879628 28305263

[B35] Lee H. O. Davidson J. M. Duronio R. J. (2009). Endoreplication: polyploidy with purpose. *Genes Dev.* 23 2461–2477. 10.1101/gad.1829209 19884253 PMC2779750

[B36] Li D. Liu Y. Pei C. Zhang P. Pan L. Xiao J. (2017). miR-285-Yki/Mask double-negative feedback loop mediates blood-brain barrier integrity in *Drosophila*. *Proc. Natl. Acad. Sci. U.S.A*. 114 E2365–E2374. 10.1073/pnas.1613233114 28265104 PMC5373330

[B37] Lilly M. A. Duronio R. J. (2005). New insights into cell cycle control from the *Drosophila* endocycle. *Oncogene* 24 2765–2775. 10.1038/sj.onc.1208610 15838513

[B38] Lilly M. A. Spradling A. C. (1996). The *Drosophila* endocycle is controlled by cyclin E and lacks a checkpoint ensuring S-phase completion. *Genes Dev*. 10 2514–2526. 10.1101/gad.10.19.2514 8843202

[B39] Liu P. C. Fu X. N. Zhu J. S. (2018). Juvenile hormone-regulated alternative splicing of the taiman gene primes the ecdysteroid response in adult mosquitoes. *Proc. Natl. Acad. Sci. U.S.A*. 115 E7738–E7747. 10.1073/pnas.1808146115 30061397 PMC6099845

[B40] Liu S. Li K. Gao Y. Liu X. Chen W. Ge W. (2018). Antagonistic actions of juvenile hormone and 20-hydroxyecdysone within the ring gland determine developmental transitions in *Drosophila*. *Proc. Natl. Acad. Sci. U.S.A*. 115 139–144. 10.1073/pnas.1716897115 29255055 PMC5776822

[B41] López-Schier H. St Johnston D. (2001). Delta signaling from the germ line controls the proliferation and differentiation of the somatic follicle cells during *Drosophila* oogenesis. *Genes Dev*. 15 1393–1405. 10.1101/gad.200901 11390359 PMC312703

[B42] Losick V. P. Jun A. S. Spradling A. C. (2016). Wound-induced polyploidization: regulation by Hippo and JNK signaling and conservation in mammals. *PLoS One* 11:e0151251. 10.1371/journal.pone.0151251 26958853 PMC4784922

[B43] Lynch M. Marinov G. K. (2015). The bioenergetic costs of a gene. *Proc. Natl. Acad. Sci. U.S.A*. 112 15690–15695. 10.1073/pnas.1514974112 26575626 PMC4697398

[B44] Mal’ceva N. I. Gyurkovics H. Zhimulev I. F. (1995). General characteristics of the polytene chromosomes from ovarian pseudonurse cells of the *Drosophila melanogaster* otu11 and fs(2)B mutants. *Chromosome Res*. 3 191–200. 10.1007/bf00710713 7780663

[B45] Maldonado K. M. Mendoza H. L. Cruz Hernandez-Hernandez F. (2019). Cell cycle dynamics and endoreplication in the mosquito midgut. *Am. J. Biomed. Sci. Res*. 5 43–46. 10.34297/ajbsr.2019.05.000871

[B46] Maqbool S. B. Mehrotra S. Kolpakas A. Durden C. Zhang B. Zhong H. (2010). Dampened activity of E2F1-DP and Myb-MuvB transcription factors in *Drosophila* endocycling cells. *J. Cell Sci*. 123 4095–4106. 10.1242/jcs.064519 21045111 PMC2987441

[B47] Mehrotra S. Maqbool S. B. Kolpakas A. Murnen K. Calvi B. R. (2008). Endocycling cells do not apoptose in response to DNA rereplication genotoxic stress. *Genes Dev*. 22 3158–3171. 10.1101/gad.1710208 19056894 PMC2593612

[B48] Meneses-Acosta A. Mendonça R. Merchant H. Covarrubias L. Ramírez O. (2001). Comparative characterization of cell death between Sf9 insect cells and hybridoma cultures. *Biotechnol. Bioeng*. 72 441–457. 10.1002/1097-0290(20000220)72:4<441::aid-bit1006>3.0.co;2-3 11180064

[B49] Moriyama M. Osanai K. Ohyoshi T. Wang H. B. Iwanaga M. Kawasaki H. (2016). Ecdysteroid promotes cell cycle progression in the *Bombyx* wing disc through activation of c-Myc. *Insect Biochem. Mol. Biol*. 70 1–9. 10.1016/j.ibmb.2015.11.008 26696544

[B50] Mottier V. Siaussat D. Bozzolan F. Auzoux-Bordenave S. Porcheron P. Debernard S. (2004). The 20-hydroxyecdysone-induced cellular arrest in G2 phase is preceded by an inhibition of cyclin expression. *Insect Biochem. Mol. Biol*. 34 51–60. 10.1016/j.ibmb.2003.09.003 14976982

[B51] Mueller P. R. Coleman T. R. Kumagai A. Dunphy W. G. (1995). Myt1: a membrane-associated inhibitory kinase that phosphorylates Cdc2 on both threonine-14 and tyrosine-15. *Science* 270 86–90. 10.1126/science.270.5233.86 7569953

[B52] Nair K. K. Chen T. T. Wyatt G. R. (1981). Juvenile hormone-stimulated polyploidy in adult locust fat body. *Dev. Biol*. 81 356–360. 10.1016/0012-1606(81)90300-6 7202845

[B53] Ohhara Y. Kobayashi S. Yamanaka N. (2017). Nutrient-dependent endocycling in steroidogenic tissue dictates timing of metamorphosis in *Drosophila melanogaster*. *PLoS Genet*. 13:e1006583. 10.1371/journal.pgen.1006583 28121986 PMC5298324

[B54] Oishi M. Locke J. Wyatt G. R. (1985). The ribosomal RNA genes of *Locusta migratoria*: copy number and evidence for underreplication in a polyploid tissue. *Can. J. Biochem. Cell Biol*. 63 1064–1070. 10.1139/o85-132 4075222

[B55] Parker L. Piwnica-Worms H. (1992). Inactivation of the p34 cdc2-cyclin B complex by the human WEE1 tyrosine kinase. *Science* 257 1955–1957. 10.1126/science.1384126 1384126

[B56] Parthasarathy R. Palli S. R. (2008). Proliferation and differentiation of intestinal stem cells during metamorphosis of the red flour beetle, *Tribolium castaneum*. *Dev. Dyn*. 237 893–908. 10.1002/dvdy.21475 18297733

[B57] Rangel J. Strauss K. Seedorf K. Hjelmen C. E. Johnston J. S. (2015). Endopolyploidy changes with age-related polyethism in the honey bee, *Apis mellifera*. *PLoS One* 10:e0122208. 10.1371/journal.pone.0122208 25881205 PMC4400096

[B58] Ray K. Mercedes M. Chan D. Choi C. Nishiura J. T. (2009). Growth and differentiation of the larval mosquito midgut. *J. Insect Sci*. 9 1–13. 10.1673/031.009.5501 20053117 PMC3011905

[B59] Ren D. Guo W. Yang P. Song J. He J. Zhao L. (2019). Structural and functional differentiation of a fat body-like tissue adhering to testis follicles facilitates spermatogenesis in locusts. *Insect Biochem. Mol. Biol*. 113:103207. 10.1016/j.ibmb.2019.103207 31421206

[B60] Ribbert D. (1979). Chromomeres and puffing in experimentally induced polytene chromosomes of *Calliphora erythrocephala*. *Chromosoma* 74 269–298. 10.1007/bf01190743 510083

[B61] Rotelli M. D. Policastro R. A. Bolling A. M. Killion A. W. Weinberg A. J. Dixon M. J. (2019). A Cyclin A-Myb-MuvB-Aurora B network regulates the choice between mitotic cycles and polyploid endoreplication cycles. *PLoS Genet*. 15:e1008253. 10.1371/journal.pgen.1008253 31291240 PMC6645565

[B62] Royzman I. Hagihara A. H. Dej K. J. Bosco G. Lee J. Y. Orr-Weaver T. L. (2002). The E2F cell cycle regulator is required for *Drosophila* nurse cell DNA replication and apoptosis. *Mech. Dev*. 119 225–237. 10.1016/s0925-4773(02)00388-x 12464435

[B63] Scholes D. R. Suarez A. V. Paige K. N. (2013). Can endopolyploidy explain body size variation within and between castes in ants? *Ecol. Evol*. 3 2128–2137. 10.1002/ece3.623 23919157 PMC3728952

[B64] Shabbeer S. Omer D. Berneman D. Weitzman O. Alpaugh A. Pietraszkiewicz A. (2013). BRCA1 targets G2/M cell cycle proteins for ubiquitination and proteasomal degradation. *Oncogene* 32 5005–5016. 10.1038/onc.2012.522 23246971 PMC3796024

[B65] Shu Z. Row S. Deng W. M. (2018). Endoreplication: the good, the bad, and the ugly. *Trends Cell Biol*. 28 465–474. 10.1016/j.tcb.2018.02.006 29567370 PMC5962415

[B66] Song J. Li W. Zhao H. Zhou S. (2019). Clustered miR-2, miR-13a, miR-13b and miR-71 coordinately target Notch gene to regulate oogenesis of the migratory locust *Locusta migratoria*. *Insect Biochem. Mol. Biol*. 106 39–46. 10.1016/j.ibmb.2018.11.004 30453026

[B67] Storchova Z. Pellman D. (2004). From polyploidy to aneuploidy, genome instability and cancer. *Nat. Rev. Mol. Cell Biol*. 5 45–54. 10.1038/nrm1276 14708009

[B68] Stormo B. M. Fox D. T. (2017). Polyteny: still a giant player in chromosome research. *Chromosome Res*. 25 201–214. 10.1007/s10577-017-9562-z 28779272 PMC5768140

[B69] Sun J. Evrin C. Samel S. A. Fernandez-Cid A. Riera A. Kawakami H. (2013). Cryo-EM structure of a helicase loading intermediate containing ORC-Cdc6-Cdt1-MCM2-7 bound to DNA. *Nat. Struct. Mol. Biol*. 20 944–951. 10.1038/nsmb.2629 23851460 PMC3735830

[B70] Sun J. J. Deng W. M. (2005). Notch-dependent downregulation of the homeodomain gene cut is required for the mitotic cycle/endocycle switch and cell differentiation in *Drosophila* follicle cells. *Development* 132 4299–4308. 10.1242/dev.02015 16141223 PMC3891799

[B71] Sun J. J. Deng W. M. (2007). Hindsight mediates the role of Notch in suppressing hedgehog signaling and cell proliferation. *Dev. Cell* 12 431–442. 10.1016/j.devcel.2007.02.003 17336908 PMC1851662

[B72] Sun J. J. Smith L. Armento A. Deng W. M. (2008). Regulation of the endocycle/gene amplification switch by Notch and ecdysone signaling. *J. Cell Biol*. 182 885–896. 10.1083/jcb.200802084 18779369 PMC2528591

[B73] Swanson C. I. Meserve J. H. McCarter P. C. Thieme A. Mathew T. Elston T. C. (2015). Expression of an S phase-stabilized version of the CDK inhibitor Dacapo can alter endoreplication. *Development* 142 4288–4298. 10.1242/dev.115006 26493402 PMC4689214

[B74] Tanaka S. Araki H. (2012). Formation of pre-initiation complex: formation of the active helicase and establishment of replication forks. *Cold Spring Harb. Perspect. Biol.* 5:a010371 10.1101/cshperspect.a010371 PMC3839609 23881938

[B75] Tang Z. Coleman T. Dunphy W. G. (1993). Two distinct mechanisms for negative regulation of the Wee1 protein kinase. *EMBO J*. 12 3427–3436. 10.1002/j.1460-2075.1993.tb06017.x 7504624 PMC413619

[B76] Taracena M. L. Bottino-Rojas V. Talyuli O. A. C. Walter-Nuno A. B. Oliveira J. H. M. Angleró-Rodriguez Y. I. (2018). Regulation of midgut cell proliferation impacts *Aedes aegypti* susceptibility to dengue virus. *PLoS Negl. Trop. Dis*. 12:e0006498. 10.1371/journal.pntd.0006498 29782512 PMC5983868

[B77] Tigan A. S. Bellutti F. Kollmann K. Tebb G. Sexl V. (2016). CDK6-a review of the past and a glimpse into the future: from cell-cycle control to transcriptional regulation. *Oncogene* 35 3083–3091. 10.1038/onc.2015.407 26500059

[B78] Unhavaithaya Y. Orr-Weaver T. L. (2012). Polyploidization of glia in neural development links tissue growth to blood-brain barrier integrity. *Genes Dev*. 26 31–36. 10.1101/gad.177436.111 22215808 PMC3258963

[B79] Van de Peer Y. Mizrachi E. Marchal K. (2017). The evolutionary significance of polyploidy. *Nat. Rev. Genet*. 18 411–424. 10.1038/nrg.2017.26 28502977

[B80] Wang D. Li X. R. Dong D. J. Huang H. Wang J. X. Zhao X. F. (2016). The steroid hormone 20-Hydroxyecdysone promotes the cytoplasmic localization of Yorkie to suppress cell proliferation and induce apoptosis. *J. Biol. Chem*. 291 21761–21770. 10.1074/jbc.m116.719856 27551043 PMC5076844

[B81] Wang Z. Yang L. Song J. Kang L. Zhou S. (2017). An isoform of Taiman that contains a PRD-repeat motif is indispensable for transducing the vitellogenic juvenile hormone signal in *Locusta migratoria*. *Insect Biochem. Mol. Biol*. 82 31–40. 10.1016/j.ibmb.2017.01.009 28137505

[B82] Welburn J. P. Tucker J. A. Johnson T. Lindert L. Morgan M. Willis A. (2007). How tyrosine 15 phosphorylation inhibits the activity of cyclin-dependent kinase 2-cyclin A. *J. Biol. Chem*. 282 3173–3181. 10.1074/jbc.m609151200 17095507

[B83] Wielgus J. J. Bollenbacher W. E. Gilbert L. I. (1979). Correlations between epidermal DNA synthesis and haemolymph ecdysteroid titre during the last larval instar of the tobacco hornworm, *Manduca sexta*. *J. Insect Physiol*. 25 9–16. 10.1016/0022-1910(79)90030-1

[B84] Wu Z. Guo W. Xie Y. Zhou S. (2016). Juvenile hormone activates the transcription of cell-division-cycle 6 (Cdc6) for polyploidy-dependent insect vitellogenesis and oogenesis. *J. Biol. Chem*. 291 5418–5427. 10.1074/jbc.M115.698936 26728459 PMC4777871

[B85] Wu Z. Guo W. Yang L. He Q. Zhou S. (2018). Juvenile hormone promotes locust fat body cell polyploidization and vitellogenesis by activating the transcription of Cdk6 and E2f1. *Insect Biochem. Mol. Biol*. 102 1–10. 10.1016/j.ibmb.2018.09.002 30205150

[B86] Xu D. Woodfield S. E. Lee T. V. Fan Y. Antonio C. Bergmann A. (2009). Genetic control of programmed cell death (apoptosis) in *Drosophila*. *Fly* 3 78–90. 10.4161/fly.3.1.7800 19182545 PMC2702463

[B87] Yamanaka N. Rewitz K. F. O’Connor M. B. (2013). Ecdysone control of developmental transitions: lessons from *Drosophila* research. *Annu. Rev. Entomol*. 58 497–516. 10.1146/annurev-ento-120811-153608 23072462 PMC4060523

[B88] Zhang B. Q. Mehrotra S. Ng W. L. Calvi B. R. (2014). Low levels of p53 protein and chromatin silencing of p53 target genes repress apoptosis in *Drosophila* endocycling cells. *PLoS Genet*. 10:e1004581. 10.1371/journal.pgen.1004581 25211335 PMC4161308

[B89] Zhang Y. Kalderon D. (2000). Regulation of cell proliferation and patterning in *Drosophila* oogenesis by hedgehog signaling. *Development* 127 2165–2176. 10769240 10.1242/dev.127.10.2165

[B90] Zhimulev I. F. (1996). Morphology and structure of polytene chromosomes. *Adv. Genet*. 34 1–490. 10.1016/s0065-2660(08)60533-7 9348397

[B91] Zhimulev I. F. Belyaeva E. S. Semeshin V. F. Koryakov D. E. Demakov S. A. Demakova O. V. (2004). Polytene chromosomes: 70 years of genetic research. *Int. Rev. Cytol*. 241 203–275. 10.1016/s0074-7696(04)41004-3 15548421

[B92] Zielke N. Edgar B. A. DePamphilis M. L. (2013). Endoreplication. *Cold Spring Harb. Perspect. Biol*. 5:a012948. 10.1101/cshperspect.a012948 23284048 PMC3579398

[B93] Zielke N. Kim K. J. Tran V. Shibutani S. T. Bravo M. J. Nagarajan S. (2011). Control of *Drosophila* endocycles by E2F and CRL4 (CDT2). *Nature* 480 123–127. 10.1038/nature10579 22037307 PMC3330263

[B94] Zielke N. Querings S. Rottig C. Lehner C. Sprenger F. (2008). The anaphase-promoting complex/cyclosome (APC/C) is required for rereplication control in endoreplication cycles. *Genes Dev*. 22 1690–1703. 10.1101/gad.469108 18559483 PMC2428065

[B95] Zimmet J. Ravid K. (2000). Polyploidy: occurrence in nature, mechanisms, and significance for the megakaryocyte-platelet system. *Exp. Hematol*. 28 3–16. 10658672 10.1016/s0301-472x(99)00124-1

[B96] Zülbahar S. Sieglitz F. Kottmeier R. Altenhein B. Rumpf S. Klambt C. (2018). Differential expression of Öbek controls ploidy in the *Drosophila* blood-brain barrier. *Development* 145:dev164111. 10.1242/dev.164111 30002129

